# An *in vitro* Survey on the Effect of Six Commercial Brands of Phosphoric Acid on the Micro-shear Bond Strength of Composite Resin to Enamel

**DOI:** 10.30476/dentjods.2023.97528.2015

**Published:** 2024-12-01

**Authors:** Faeze Jamali Zavare, Mahsa Sheikholeslamian, Saber Kazemi, Amir Ghasemi, Narges Panahandeh, Mina Yazdizadeh, Elham Zanguei

**Affiliations:** 1 Dept. of Restorative Dentistry, Dental Research Center, School of Dentistry, Shahid Beheshti University of Medical Sciences, Tehran, Iran; 2 Dept. of Restorative Dentistry, Dental Research Center, School of Dentistry, Tehran University of Medical Sciences, Tehran, Iran; 3 Dentist, Dental Research Center, Dept. of Restorative, School of Dentistry, Shahid Beheshti University of Medical Sciences, Tehran, Iran; 4 Dept. of Pediatric Dentistry, Dental Research Center, School of Dentistry, Birjand University of Medical Sciences, Birjand, Iran; 5 Dept. of Restorative Dentistry, Dental Research Center, School of Dentistry, Birjand University of Medical Sciences, Birjand, Iran

**Keywords:** Composite resins, Dental acid etching, Dental bonding, Dental Enamel, Shear strength

## Abstract

**Statement of the Problem:**

One of the most important factors in the clinical longevity of composite resin restorations is proper adhesion, which is achieved using phosphoric acid. Different phosphoric acid products might affect the micro-shear bond strength of composite resin to enamel.

**Purpose:**

The present study aimed to evaluate the micro-shear bond strength of composite resin to sound enamel using six different brands of acid-etch agents.

**Materials and Method:**

The present *in vitro* study was carried out on 72 extracted sound human (mandibular and maxillary) first premolar teeth. The teeth crowns were divided into two equal parts with a mesiodistal cut and randomly assigned to six groups in terms of the acid etchant brand: Ultra-etch, SDI, Morvabon, FGM, Nik Darman, and Experimental. The bonding procedure was carried out using the Margin Bond enamel adhesive. Tygon tubes (1×0.7 mm) containing composite resin were bonded to each enamel surface. After 24 hours of storage in distilled water, each sample underwent a shearing force with a crosshead speed of 0.5 mm/min. The morphologic changes were evaluated using scanning electron microscopy. The data were analyzed with SPSS using one-way ANOVA and Tukey test (*p*< 0.05).

**Results:**

One-way ANOVA showed that micro-shear bond strength in different etchant agents have significant differences (*p*< 0.001). The highest and lowest composite resin micro-shear bond strengths belonged to Ultradent and SDI brands, respectively. The enamel surfaces in the SDI group revealed residual debris after dissolution.

**Conclusion:**

Preparation of the enamel surface with different phosphoric acid products might affect the micro-shear bond strength and enamel surface morphology differently. Further clinical studies are suggested to evaluate the effect of different types and concentrations of acid etching agents on the enamel and dentin bonds.

## Introduction

The clinical success of composite resin restorations mostly depends on the efficacy and quality of the bonding system used, which provides a durable and effective bond between composite resin and tooth structure. The effective bond decreases marginal microleakage, bacterial penetration, recurrent caries, and pulpal sensitivity and inflammation [ 1 ]. 

Adhesion is largely determined by the characteristics of the interfacing surfaces and the properties of the material used as a bonding agent [ 2 ]. Etching with phosphoric acid will lead to adhesion of bonding agent to enamel, resulting in a durable bond [ 3 ]. The mechanism of adhesion involves the formation and penetration of resin tags into enamel microporosities resulting from the dissolution of hydroxyapatite crystals by the acid etch agent [ 4 ]. The resin tags within the enamel microporosities form a micromechanical interlocking at the enamel bonding agent interface [ 4 ].

Enamel and dentin have different structures and compositions. Therefore, their adhesion properties and mechanisms are different from each other [ 5 ]. The enamel surface morphology can be changed more easily by using phosphoric acid etching, which has a main mineral content of up to 96%. Moreover, enamel-etching results in a significant increase in surface energy, which is beneficial for achieving satisfactory enamel [ 6 ]. The surface contour can be modified by the acid etch in the dental enamel by cleaning superficially and removing the smear layer. Tooth hydroxyapatite crystals are dissolved by acid etch and surface energy is increased, and more moisture is obtained due to a smaller contact angle of the adhesive with the tissue. Acid etch reacts with the release of carbon and the separation of calcium and phosphorus, leading to irregularities in the intra and inter crystalline [ 6 - 10 ]. Under such condition, if a resin-based material with fluid properties, without fillers, and with low viscosity (enamel bonding agent) is placed on the etched irregular surface, the resin penetrates the micrometer porosities, which is boosted by the capillary effect [ 11 ]. The monomers are polymerized, and the material is located within the enamel surface, forming resin tags [ 11 - 14 ]. According to Gwinnett and Garcia-Godoy [ 15 ], these resin tags are the main factors for adhesion to the enamel surface by creating a micromechanical bond. Currently, 30‒40% (usually 37%) phosphoric acid is used to etch the enamel surface [ 12 ]. The duration of etching the enamel surface with 30‒40% phosphoric acid is 60 seconds. Although only one study reported that shorter etching times result in lower bond strength, many studies have shown that etching for 15 seconds provides a rough surface similar to that obtained from etching for 60 seconds [ 12 , 16 - 17 ]. The suggested etching time for permanent and primary enamel using Bis-GMA-based adhesive systems with 32-40% phosphoric acid is 15 seconds [ 7 , 18 ]. Although it has been shown that the shear bond strength to enamel is not compromised by reducing acid etching time and bond strength of various acid etching times has not shown any statistically significant differences in many studies [ 19 - 20 ]. Acid etching for enamel has advantages that include improving the wettability of the surface [ 21 ], surface roughness [ 22 ], and surface free-energy [ 23 ], and improving bonding, despite the enamel's decreased surface hardness [ 9 ]. Gu *et al*. [ 24 ] investigated the effects of etching with different concentrations of phosphoric acid on the micro tensile bond strength of Adper Single Bond 2 (3M ESPE, St Paul MN, USA) to teeth with fluorosis. They reported that phosphoric acid concentration significantly affected the micro tensile bond strength of Adper Single Bond 2 to the enamel. The maximum bond strength was achieved with 40% phosphoric acid. Generally, two factors affect the favorable bond between the enamel and bonding agents which includes adequate penetration of the bonding agent into the demineralized enamel that depends on the wetting between the adhesive system and enamel [ 13 ] and the properties (strength) of the resin bonding agent [ 13 ].

Different techniques are available to determine the bond strength of different bonding agents. Although micro-tensile bond strength tests can measure adhesive bond strength, the Academy of Dental Materials maintains that shear bond strength tests are appropriate for measuring the adhesion on enamel [ 25 ], and this method is well-known and established [ 26 - 27 ]. The major advantage of this test over micro tensile bond strength tests is that the samples are pre-stressed before the test by eliminating the mold [ 27 ]. Better adhesion to the tooth structure might help the retention of the restoration, decreasing the need for mechanical retention features involving the preparation of the sound tooth structure and being effective in decreasing microleakage and its consequences [ 28 ]. In addition, the establishment of a bond at the tooth‒restoration interface counteracts the forces resulting from polymerization shrinkage; which is considered as one of the factors involved in preserving the integrity of the margins [ 28 ]. The present study aimed to evaluate the effect of six commercial brands of phosphoric acid on the micro shear bond strength of composite resin to the enamel. 

## Materials and Method

This study was approved by the Human Ethics Committee of Shahid Beheshti University of Medical Sciences (IR.SBMU.RIDS.1394.), Tehran, Iran, and complied with the ethical guidelines of the *Helsinki Declaration*.

### Sample size and preparation

Considering previous studies on the shear bond strength [ 29 - 30 ], 12 samples were included in the study for each phosphoric acid brand. To minimize the confounding effect of fluoride content differences in various types of teeth, only first premolars were considered in this study [ 31 ]. 72 sound (mandibular and maxillary) premolar teeth with no crack/fracture, decay, erosion and hypoplastic defects which were extracted for orthodontic reasons were collected, cleaned of all the calculi and soft tissues with periodontal curettes, and used in this study. They were disinfected in a 0.5% Timol solution and stored in 1% normal saline solution at room temperature until use [ 6 , 32 - 36 ]. Then, the samples were cut mesiodistally using a thin-sectioning machine (Hamco Machines Inc. Rochester, NY,USA) into two almost equal halves and prepared for the test [ 32 , 35 , 37 ]. To obtain a flat, smooth surface with a standard smear layer, surfaces of the enamel were ground with wet 400-, 600-, 800-, 1200-, 1500-, 1800-, and 2000- grit polishing papers [ 35 , 37 - 41 ]. Finally, the samples were randomly assigned to six groups (n=12) in terms of the acid etching agent brand to prepare the enamel surface. The etching step was carried out with six brands of phosphoric acid gels (SDI(Super Etch, Southern Dental Industries- SDI, Bayswater, Victoria 3153, Australia), Morvabon (Morva Etchant; Tehran, Iran), FGM (Condac 37; FGM, Joinville, SC, Brazil), Ultradent (Ultra-etch, Ultradent, South Jordan, UT, USA), Nik Darman (Etch one, Nik Darman, Tehran, Iran), and Experimental) for 20 seconds[ 29 ], followed by irrigation with air and water syringe for 40 seconds.

### The bonding and composite resin placement process

The bonding procedures were carried out with the Margin Bond (Coltene Whaledent, USA) adhesive. The first layer of the bonding agent was applied with a micro brush according to the manufacturers’ instructions, followed by applying a mild air stream to evaporate its volatile components. Then light-curing was carried out with a light-curing unit (Kerr Orange, CA, USA) at 550mW/cm^2^ light intensity. Tygon tubes, measuring 0.7 mm in internal diameter and 1 mm in height, were prepared to place composite resin. First, the tubes were fixed on the prepared surface. Then, Filtek Z250 (3M ESPE, St. Paul, MN, USA) composite resin was placed within the tubes and light-cured for 40 seconds [ 6 , 32 , 38 ]. The samples were stored at room temperature before removing the Tygon tubes, which were cut with a scalpel blade and removed. Then the samples were immersed in distilled water and incubated at 37ºC for 24 hours. The universal testing machine (Unitek 94100) was used to test the shear bond strength of samples at a crosshead speed of 0.5 mm/min using a wedge-shaped blade with a surface of 0.2 mm until failure. The force necessary for debonding was determined, and the bond strength was calculated in MPa [ 42 ].

Subsequently, failure modes of all the samples were examined using a scanning electron microscope (SEM) (Hitachi SU 3500, Japan). Samples were prepared for SEM evaluations at ×400 and ×2000 magnifications, using secondary electric detectors (to evaluate the surface morphology and microstructure) and electron backscattering (to separate phases based on atomic number difference) at 10 kV. SEM micrographs were prepared to evaluate the patterns created by acid etching and its comparison with internationally accepted standards [ 43 ].

### Statistical analysis

The variables were analyzed with SPSS 20. Means and standard deviations were used to describe the mean micro shear bond strength in each group. The normal distribution of data was analyzed with the Kolmogorov-Smirnov test. One-way ANOVA and Games-Howell test were used to compare the groups. Statistical significance was set at *p*< 0.05.

## Results

**Figure 1:**
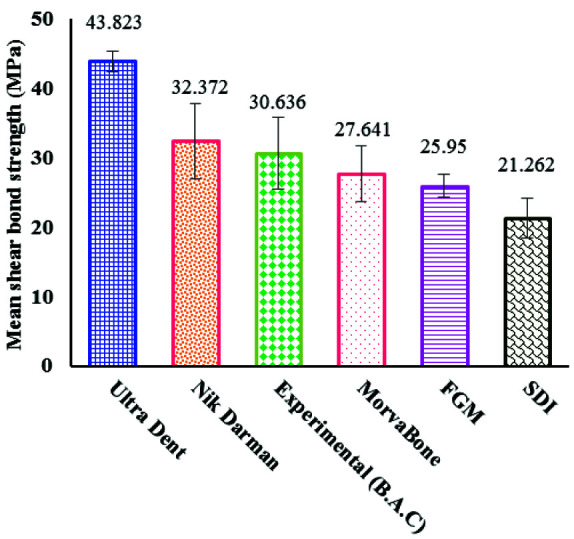
The means (SD) of micro-shear bond strength of composite resin to enamel after preparation with Ultradent (Ultra-etch, Ultradent, South Jordan, UT, USA); SDI (Super Etch, Southern Dental Industries – SDI, Bayswater, Victoria 3153, Australia); Morvabon (Morva Etchant; Tehran, Iran); FGM (Condac 37; FGM, Joinville, SC, Brazil); Nik Darman (Etch one, Nik Darman, Tehran, Iran); Experimental

Comparison of the mean micro-shear bond strength of composite resin to enamel after preparation with different brands of phosphoric acid gel with one-way ANOVA showed significant differences between the different brands (*p*< 0.001). The means and standard deviations of micro-shear bond strength of composite resin to enamel in descending order were as follows: Ultradent, Nik Darman, Experimental, Morvabon, FGM, and SDI (Table 1, Figure 1).

**Table 1:** The means and standard deviations of micro-shear bond strength of composite resin to enamel after preparation with Ultradent (Ultra-etch, Ultradent, South Jordan, UT, USA); SDI (Super Etch, Southern Dental Industries – SDI, Bayswater, Victoria 3153, Australia); Morvabon (Morva Etchant; Tehran, Iran); FGM (Condac 37; FGM, Joinville, SC, Brazil); Nik Darman (Etch one, Nik Darman, Tehran, Iran); Experimental phosphoric acids

Phosphoric acid brands	Micro-shear bond strength Mean±SD (MPa)	Minimum	Maximum
Ultradent	43.823±1.481	41.92	47.13
Nik Darman	32.372±5.451	23.95	41.14
Experimental	30.636±5.224	23.43	36.97
Morvabon	27.641±3.977	21.09	35.93
FGM	25.950±1.704	23.17	28.90
SDI	21.262±2.824	16.92	26.82

### Two-by-two comparison of mean micro-shear bond strengths of composite resin to enamel after preparation with different brands of phosphoric acid gel based on Games-Howell test

The highest mean micro-shear bond strength value was achieved with the Ultradent phosphoric acid gel, which was significant compared to the SDI brand (*p*= 0.007). However, there were no significant differences between this brand and the rest of the brands. The lowest mean micro-shear bond strength of composite resin to enamel was achieved with the SDI phosphoric acid gel, which was significant compared to FGM (*p*= 0.003), Morvabon (*p*= 0.011), Nik Darman (*p*= 0.001), Ultradent (*p*= 0.007), and Experimental (*p*= 0.004) brands. There were no significant differences between the FGM and Morvabon (*p*= 0.928), Nik Darman (*p*= 0.121), Ultradent (*p*= 0.137), and Experimental (*p*= 0.866) phosphoric acid gel brands. There were no significant differences between Morvabon phosphoric acid gel and Nik Darman (*p*= 0.635), Ultradent (*p*= 0.378), and Experimental (*p*= 0.802) phosphoric acid gel brands. There were no significant differences between Nik Darman phosphoric acid gel and Ultradent (*p*= 0.943) and Experimental (*p*= 0.802) phosphoric acid gel brands. There was no significant difference between Ultradent phosphoric acid gel and Experimental phosphoric acid gel (*p*= 0.487).

### Determination of enamel surface micromorphology after preparation with different phosphoric acid gel brands

**Figure 2:**
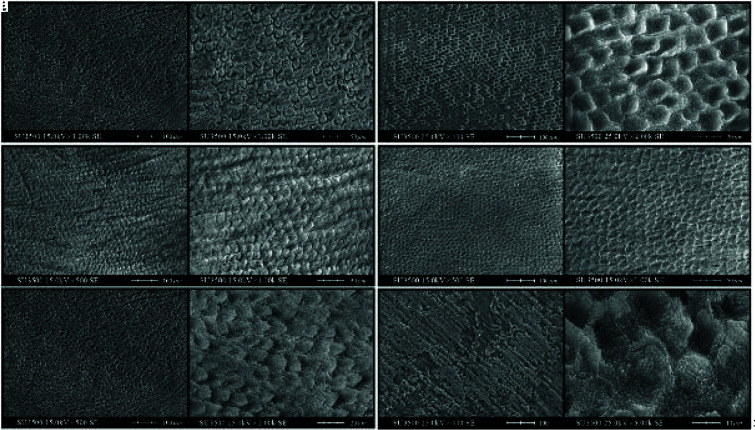
SEM image after etching in different magnifications with **a:** Ultradent phosphoric acid gel(Ultra-etch, Ultradent, South Jordan, UT, USA), **b:** Nik Darman phosphoric acid gel(Etch one, Nik Darman, Tehran, Iran), **c:** Experimental phosphoric acid gel, **d:** Morvabon phosphoric acid gel(Morva Etchant; Tehran, Iran), **e:** FGM phosphoric acid gel(Condac 37; FGM, Joinville, SC, Brazil), **f:** SDI phosphoric acid gel(Super Etch, Southern Dental Industries – SDI, Bayswater, Victoria 3153, Australia)

Based on SEM Images, the maximum debris was observed with the SDI phosphoric acid gel. According to SEM images, the etching patterns of different phosphoric acid gel brands were as Figure 2.

### Determination of the failure of the bond of composite resin to enamel after preparation with different phosphoric acid gel brands

Table 2 presents the failure modes after the micro-shear bond strength test. In all the samples, a combination of mixed, cohesive, and adhesive failure modes was observed. Cohesive failure was predominant with the Ultradent brand. Adhesive failure was more common with the SDI brand than other brands (Table 2).

**Table 2:** The failure modes of Ultradent (Ultra-etch, Ultradent, South Jordan, UT, USA); SDI (Super Etch, Southern Dental Industries- SDI, Bayswater, Victoria 3153, Australia); Morvabon (Morva Etchant; Tehran, Iran); FGM (Condac 37; FGM, Joinville, SC, Brazil); Nik Darman (Etch one, Nik Darman, Tehran, Iran); Experimental phosphoric acids after the micro-shear brand strength test

Phosphoric acid brands	Failure mode		
	Adhesive	Cohesive	Mixed
Ultradent	8.3%	58.3%	33.4%
Nik Darman	25%	33.3%	41.7%
Experimental	25%	25%	50%
Morvabon	16.7%	33.3%	50%
FGM	16.7%	25%	58.3%
SDI	33.3%	25%	41.7%

## Discussion

The present study aimed to evaluate the effect of six different brands of phosphoric acid gel on the micro-shear bond strength of composite resin to the enamel. Using phosphoric acid for bonding composite resin to tooth structure is one of the most sensitive steps in the bonding process [ 29 ]. In the enamel bond process, acid etching increases the surface energy. Resin placed on the surface of the enamel penetrates into the etched enamel with the help of the capillarity of the resin, and resin tags are formed after its polymerization [ 44 ]. In this study, the micro-shear bond test is used to evaluate the bond strength of composite resin to enamel. The micro-shear bond strength test is widely used due to the ease of the test and preparation of the samples, with easy instructions and reliability. The micro-shear bond strength is a useful tool to understand the complexity of the interaction between dental composite resins, dentin, and enamel at the bonding interface; the macro-shear bond strength test cannot evaluate such interactions [ 45 ].

The results of the current study shows that the means and standard deviations of micro-shear bond strength of composite resin to enamel are as follows in descending order: Ultradent (43.823 E1,481), Nik Darman (32.372±5.451), Experimental (30.636±5.224), Morvabon (27.641±3.977), FGM (25.950±1.704), and SDI (21.262±2.824). It means that etching acids are different and some of them used in this test have higher mean bond strength values compared to others etchants.

The shear bond strength of composite to etched phosphoric acid enamel is usually higher than 20 MPa and can be more than 50 MPa depending on the type of test that evaluates the bond strength [ 46 ]. Clinically, an average bond strength of 20 MPa appears to be acceptable for enamel [ 44 ]. In the present study, the average shear band strength values of all brands are higher than this value and it seems that adhesion is clinically acceptable.

Regarding to the study that Shafiee *et al*. [ 13 ] carried out on composite shear bond strength to dry and wet enamel using 3M phosphoric acid brand (37%), shear bond strength of 20.99 MPa was reported, which is lower than all etchants tested in this research. Munari *et al*. [ 30 ] reported 6.27 MPa as the average micro tensile bond strength of FGM acid brand which is lower than the value obtained in the present study; it could be due to the different type of adhesive(Single Bond 2)and glass ionomer used.

Najafi-Abrandabadi *et al*. [ 47 ] reported the value 25.64 MPa for the micro shear bond strength of enamel samples after the application of SDI phosphoric acid with a Margin Bond that is similar to the present study. Moghadam *et al*. [ 48 ] evaluated the shear band strength of 4 brands of acid etch including Kimia(Aghel manesh, Tehran, Iran), Morvabon (Morva Etchant; Tehran, Iran), Ultra Etch (Ultradent, South Jordan, UT, USA), and Etch Rite(Pulpdent Corp., Watertown, Massachusetts, USA). They observed the highest shear bond strength in the Morvabon brand and no significant difference among the other three brands. The results and study method were different from the present study.

Shear strength of etched enamel surface depend on many factors such as enamel etching pattern, acids type, acid concentration, and etching time [ 49 ]. Since the etching time is the same in all groups, we will review the other factors.

Topographically, histological characteristics called etching patterns emerge on etched tooth enamel after acid etching. Enamel's bonding receptivity is largely determined by the extent and depth of the etching pattern produced by etchants, which provides micro-mechanical interlocking. [ 50 ]. Moghaddam *et al*. [ 29 ] evaluated samples up to the etching step using SEM images of the samples etched with Ultradent phosphoric acid gel, concluding that a specific etching pattern was created on the enamel surface with this brand. In contrast, the Morvabon acid etch brand created such a pattern at a much lower rate, with the scattered and irregular orientation of the etched enamel prisms.

 Formation of deposits is another reason for the difference of shear bond strength of different brands [ 29 ]. It was shown in a study that in the Ultradent brand, there was a limited amount of deposits, or they were absent. However, in areas etched with Morvabon acid etch brand, the products resulting from acid corrosion were visible in many areas [ 29 ]. Since the highest debris was observed with the SDI brand based on SEM images and one of the surface preparation steps after applying acid is continuous irrigation, it can be concluded that possibly the highest amount of remaining debris with this brand is the reason for a lower bond strength with this brand than other brands. Despite of complete rinsing, some deposits may still be trapped in the enamel surface pores, making it impossible for adhesive to penetrate, so it will decrease the adhesive and consequently reduce the formation of resin tags [ 51 ]. The etching pattern could result in the entrapment of phosphoric acid components that are not removed by the established rinsing time [ 6 ]. This phenomenon can explain why there are more adhesive failures in the SDI brand than in the other sub groups.

It appears that acid etching of the enamel improves retention by the selective erosion of hydroxyapatite and facilitation of penetration by improving resin tags to a length of 6‒12 mm [ 52 ]. In addition, the surface of the matrix has an important role in increasing the bond strength because an increase in the roughness of the surface increases the free energy of the surface and the wettability of the adhesive system [ 53 ]. In this context, the 35% Ultradent acid etch exhibited a better performance despite a lower concentration than other brands and could create better micromechanical retention in enamel microporosities because of more surface roughness that resulted in higher bond strength.

The results achieved can also be related to the composition of acids and even the elimination of the thickener on the dental surface. Bernales Sender *et al*. [ 6 ] evaluated the influence of various phosphoric acids before application of universal adhesive on the dental enamel. They reported that mean bond strength of Condac 37 acid, which was composed, of 37% phosphoric acid gel, dye, deionized water, and a thickener was lower than others. It was believed that the thickener would not be totally removed during the established rinsing time, and therefore, adequate diffusion of monomer in inter- and intra- prismatic areas is prevented. 

Contradictory findings have been reported concerning acid concentration. Gu *et al*. [ 24 ] evaluated the microtensile bond strength of sound enamel, reporting that a decrease in phosphoric acid concentration from 45% to 40% and 35% increased the bond strength. Niaki *et al*. [ 54 ] investigated the effect of phosphoric acid concentration on the tensile bond strength of metallic brackets, reporting that decreasing the phosphoric acid concentration from 37% to 15% increased the bond strength. Some studies have reported that phosphoric acid concentration does not affect the bond strength. Legler *et al*. [ 55 ] evaluated the effects of 5%, 15%, and 37% phosphoric acid on the bond strength of orthodontic brackets, reporting that phosphoric acid concentration did not affect the tensile bond strength. Gwinett *et al*. [ 15 ] investigated the enamel bond strength of maxillary deciduous central incisors, reporting no significant difference between the 37% and 10% concentrations of phosphoric acid in bond strength. Guedes *et al*. [ 56 ] investigated the microtensile bond strength of sound enamel with 35%, 45%, and 55% concentrations of phosphoric acid. They reported that increasing the concentration of phosphoric acid did not affect the bond strength.

 To achieve adequate demineralization and etching patterns, it is recommended to use phosphoric acids in concentrations of 30% to 40% [ 6 , 57 - 59 ], and the concentration of etchants used in this study was in this range. Since the bond strength after etching with 35% Ultradent phosphoric acid was higher than that in other groups with 37% concentration, it does not seem the minor difference in concentration of phosphoric acids used could affect the bond strength in the present study.

According to SEM images, demineralization results in microporosities that raise the surface roughness index and the geometric surface of the enamel in each group. It seems all the three etching patterns were observed on the enamel surface after preparation with different phosphoric acid gel brands. Tooth enamel surfaces etched with different phosphoric acids with the exception of group SDI showed features that looks like type II etching patterns in which demineralization occurs in the neck or caudal end of the crystal [ 32 , 60 - 61 ]. Since almost the same etching pattern come up in most groups; therefore, insignificant difference of micro-shear bond strength is justifiable. Type III etching pattern defined by irregular areas without a distinct pattern that decrease the depth and increase the amplitude of the micropores [ 61 ] is limited to the surface of the dental enamel that has been etched with SDI acid. Lower values of bond strength in SDI group can be related to its irregular etching pattern and highest debris observed with the SDI brand based on SEM images [ 6 ]. 

Failures of adhesive joints occur in three locations and they are classified as adhesive, cohesive, and mixed types. Adhesive failure reveals no evidence of enamel fracture or composite resin residue on the tooth; cohesive failure shows entire fracture of enamel or resin, and mixed failures present both adhesive and cohesive failures [ 62 ]. Those groups who have more mixed and cohesive failures, as shown by the analysis of the failure mode and shear bond strength, have better bonding than those with more adhesive failures. The relation between shear bond strength and failure mode is that the cohesive failure of composite is always correlated with high shear bond strength values. Moreover, the mixed failure is more favorable than the adhesive failure, which means that if the bond strength of an adhesive system is higher, the mode of failure will usually be cohesive instead of adhesive [ 63 - 64 ]. This matter is in agreement with the findings of this research, showing that Ultradent brand with higher bond strength is the brand that exhibited the most cohesive failures and the lowest percentage of adhesive failures. Adhesive failures were most prevalent in SDI brand, which had the lowest bond strength. The results presented are similar to those reported in previous study [ 65 ]. 

Finally, it should be noted that the phosphoric acid brand affected the bond strength, and the clinical and laboratory conditions are different from each other. The present study was *in vitro*, and it is necessary to note that the temperature, moisture, and other conditions in the oral cavity are very different from laboratory conditions. In the present study, the enamel surface constituted the bonding surface. Therefore, a hydrophobic bonding agent was used, and an increase in the bond strength of hydrophilic bonding agents used on dentin was not necessarily a consideration [ 66 ]. 

Finally, the null hypothesis of the present study was refuted because the commercial brand of phosphoric acid affected the micro-shear bond strength of composite resin to enamel and the enamel surface micromorphology. 

The *in vitro* nature of the research can be considered as one of the limitations of the study. Further clinical studies are necessary to evaluate the effect of different types and concentrations of acid etching agents on the enamel and dentin bonds. Since the viscosity of etchant can influence the bond strength and etchants have different viscosities, measurement of the viscosity of etchants for better comparison is recommended in future studies. In addition, evaluation of the bond strength with SEM and other methods is suggested. 

## Conclusion

The phosphoric acid brand can affect the enamel bond strength and the surface micromorphology.

The lowest micro-shear bond strength was achieved with the SDI brand, with a significant difference from other brands. The bond strength in the Experimental group was not significantly different from the Nik Darman, Ultradent, Morvabon, and FGM groups. 

The most frequent failure mode in the Nik Darman, Experimental, Morvabon, and FGM groups was mixed, with cohesive type in the Ultradent and adhesive type in the SDI phosphoric acid groups.
